# Foretinib inhibits angiogenesis, lymphangiogenesis and tumor growth of pancreatic cancer *in vivo* by decreasing VEGFR-2/3 and TIE-2 signaling

**DOI:** 10.18632/oncotarget.3613

**Published:** 2015-04-01

**Authors:** Hsiu-Mei Chen, Chia-Hua Tsai, Wen-Chun Hung

**Affiliations:** ^1^ National Institute of Cancer Research, National Health Research Institutes, Tainan 704, Taiwan, Republic of China

**Keywords:** foretinib, c-MET, vascular growth factor receptor, TIE-2, LYVE-1

## Abstract

Foretinib, a multiple kinase inhibitor undergoing clinical trials, could suppress the activity of hepatocyte growth factor (HGF) receptor c-MET and vascular endothelial growth factor receptor-2 (VEGFR-2). In addition, Foretinib may inhibit two critical lymphangiogenic signaling receptors VEGFR-3 and TIE-2. However, the effect of Foretinib on lymphatic endothelial cells (LECs) *in vitro* and lymphangiogenesis *in vivo* is still unknown. We found Foretinib decreased basal- and HGF-induced c-MET activity at low concentrations. However, Foretinib only reduced the proliferation of pancreatic cancer cells at high concentration reflecting the intrinsic chemoresistance of pancreatic cancer cells. Foretinib inhibited VEGF-A, VEGF-C and Angiopoetin-2 (ANG-2)-stimulated tube formation and sprouting of LECs by reducing VEGFR-2, VEGFR-3 and TIE-2 activation and increased apoptosis of LECs. In xenograft animal study, Foretinib suppressed tumor growth by inhibiting proliferation, angiogenesis and lymphangiogenesis. Additionally, Foretinib inhibited angiogenesis and lymphangiogenesis more significantly and exhibited low detrimental effect in orthotopic animal study. Collectively, we suggested that Foretinib simultaneously inhibits cancer cells and LECs to reduce pancreatic tumor growth *in vivo* and demonstrated for the first time that Foretinib suppresses angiogenesis and lymphangiogenesis by blocking VEGFR-2/3 and TIE-2 signaling.

## INTRODUCTION

Foretinib was developed as an ATP-binding site competitor to inhibit receptor tyrosine kinases (RTKs) [1]. *In vitro* kinase inhibition profile showed that Foretinib suppressed c-MET, RON, VEGFRs, c-KIT, FLT-3, platelet-derived growth factor receptors (PDGFRs) and TIE-2 while it exhibited less effect on fibroblast growth factor receptor 1 (FGFR1) and epidermal growth factor receptor (EGFR). Because Foretinib is a potent inhibitor of c-MET, this drug suppressed different c-MET-activated cell lines and reduced tumor growth in different animal studies [2–4]. Accumulating evidence indicated that Foretinib targeted additional kinases to reduce tumor growth. For example, Foretinib suppressed c-JUN N-terminal kinase (JNK) to induce mitotic catastrophe in chronic myelogenous leukemia cells [5] and inhibited the TAM family of RTKs including AXL, TYRO3 and MER to kill glioblastoma cells [6]. Recently, Foretinib was shown to be a potent inhibitor of ROS1 fusion oncogenes which occurred in lung adenocarcinoma, cholangiocarcinoma and glioblastoma [7].

Pancreatic cancer is one of the lethal malignancies in the world [8]. The mean survival is around 6 months, and 5-year overall survival rate is less than 4% of the patients [9]. Pancreatic cancer is highly refractory to treatment despite numerous ongoing clinical trials using new agents and treatment combinations. The anti-cancer effect of Foretinib has been studied in pancreatic islet cancer [10]. Foretinib disrupted tumor vasculature, reduced pericyte association and emptied basement membrane sleeves which led to intratumoral hypoxia and cancer cell apoptosis. In addition, Foretinib directly inhibited pancreatic islet cancer cells and suppressed tumor metastasis to the liver. Over-expression of HGF and c-MET was detected in a high proportion of pancreatic cancer [11, 12]. In addition, expression of RON kinase, another target of Foretinib, was also up-regulated in pancreatic cancer [13]. However, the effect of Foretinib on pancreatic cancer is still unclear.

As a potent inhibitor of VEGFRs, Foretinib is expected to exhibit anti-angiogenic activity *in vivo*. Previous studies indeed demonstrated the inhibition of tumor angiogenesis by Foretinib in experimental animals. However, it remains unaddressed whether Foretinib could inhibit tumor lymphangiogenesis. Lymphatic invasion is a critical route for many cancers to spread to distant organs. Tumor cells secreted pro-lymphangiogenic factors to stimulate the proliferation of LECs and promoted the formation of new lymphatic vessels (known as lymphangiogenesis) for invasion [14].

The VEGF family consists of five members VEGF-A, -B, -C, -D and placenta growth factor (PLGF). These growth factors receive proteolytic processing *in vivo* to generate different isoforms which bind three cognate receptors VEGFR-1, VEGFR-2 and VEGFR-3 to elicit their biological function [15]. VEGFR-1 known to be activated by VEGF-A, VEGF-B and PLGF is expressed in vascular endothelial cells (VECs) [16–18]. VEGFR-2 bound by VEGF-A is expressed in both VECs and LECs and is implicated in different aspects of endothelial cell function [14]. VEGFR-3 is mainly expressed in LECs and is one of the major receptors involved in the development of lymphatic system. VEGF-C and VEGF-D bind VEGFR-3 to activate downstream ERK and AKT signaling to stimulate the proliferation of LECs. In addition to the VEGFR signaling, the angiopoietin (ANG)/TIE-2 pathway is important in lymphatic development and tumor lymphangiogenesis [19]. ANG-1 and ANG-2 can activate TIE-2 to enhance survival, growth and migration of LECs under various physiological and pathological circumstances [20]. Because Foretinib inhibits both VEGFR-3 and TIE-2, it is possible that Foretinib can block tumor lymphangiogenesis.

In this study, we investigated (1) the cytotoxic effect of Foretinib on pancreatic cancer cells, (2) the effect of Foretinib on the growth and function of LECs *in vitro* (3) the anti-cancer and anti-lymphangiogenic activity of Foretinib *in vivo*.

## RESULTS

### Foretinib inhibits the c-MET signaling of pancreatic cancer cells at low concentration while it only decreases viability of pancreatic cells at high concentration

**Figure 1:**
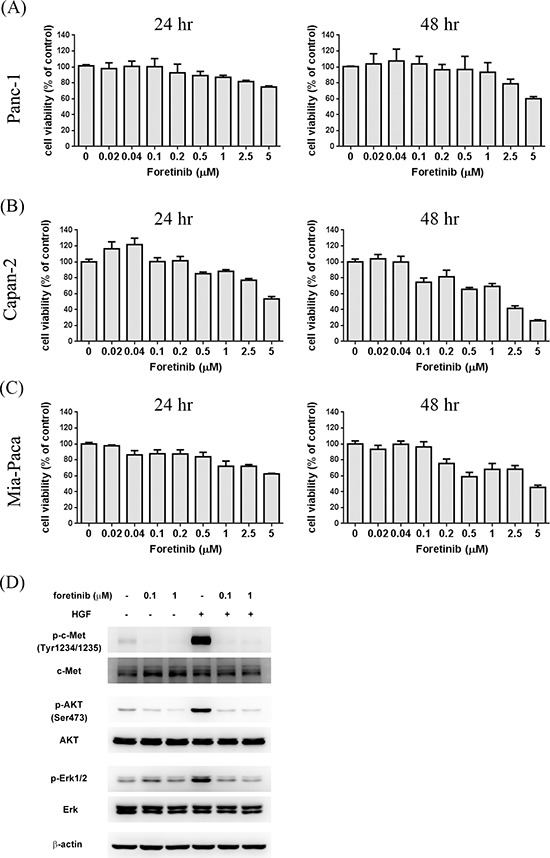
Effect of Foretinib on viability of pancreatic cancer cell lines and the activity of c-Met, AKT and ERK Human pancreatic cancer cell lines Panc-1 **A.** Capan-2 **B.** and Mia-PaCa **C.** were treated with indicated concentrations of Foretinib in growth medium containing 10% FBS for 24 or 48 h and the cell viability was measured. Values were Mean ± SEM of three separate experiments. **D.** Panc-1 cells were pre-treated with indicated concentrations of Foretinib for 1 h and then co-incubated with or without HGF (50 ng/ml) for 15 min. Cellular proteins were separated by SDS-PAGE and the blots were probed with different antibodies to detect the total or phosphorylated form of c-MET, AKT, ERK. β-actin was used as the sample loading control.

To investigate anti-cancer activity of Foretinib, the viability of three different pancreatic cancer cell lines after Foretinib treatment was studied. Viability was only slightly reduced by 1 μM of Foretinib in Panc-1, Capan-2 and Mia-Paca cells (Fig. 1A–1C). Foretinib at 5 μM showed significant cytotoxic effect on Capan-2 cells (Fig. 1B) while less effect was found in Panc-1 and Mia-Paca cells. Next, we used non-cytotoxic concentrations (0.1 and 1 μM) of Foretininb to treat Panc-1 cells and studied its effect on the activation of c-MET and downstream signaling pathways. Hepatocyte growth factor (HGF) increased phosphorylation of c-MET and its downstream AKT and ERK which could be significantly inhibited by Foretinib (Fig. 1D). These data indicated that Foretinib is a potent inhibitor of c-MET in pancreatic cancer as found in other cancers. However, Foretinib only showed significant anti-cancer activity at high concentration reflecting the intrinsic chemoresistance of pancreatic cancer cells and additional RTKs beside c-MET are important for the proliferation and survival of these cancer cells.

### Reduction of growth factor-induced tube formation and spheroid sprouting of LECs by Foretinib is associated with inhibition of VEGFR-2, VEGFR-3 and TIE-2 signaling

**Figure 2:**
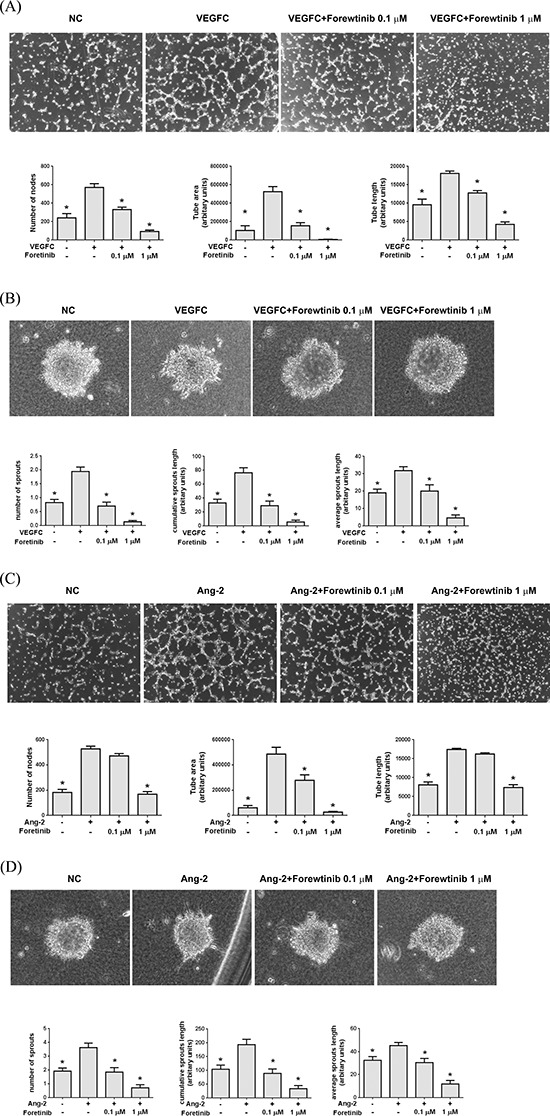
Foretinib inhibits VEGF-C and ANG-2-induced tube formation and spheroid sprouting of LECs **A.** Tube formation assay was done as described in Materials and Methods. LECs were pre-treated with indicated concentrations of Foretinib for 20 min, stimulated with VEGF-C and seeded on pre-coated Matrigel for 4 h. The number of nodes, tube area and tube length formed by LECs was measured. Values were Mean ± SEM of at least three separate experiments. **B.** The LEC spheroids were suspended in collagen gel and incubated in medium containing indicated concentrations of Foretinib and co-incubated with VEGF-C for 24 h. The number of sprouts, cumulative sprouting length and average sprouting length was measured. Values were Mean ± SEM of four separate experiments. **C.** Effect of Foretinib on ANG-2-induced tube formation was assayed as described in (A). **D.** Effect of Foretinib on ANG-2-induced tube formation was assayed as described in (B). Statistical analysis was determined by One-Way ANOVA, followed by Dunnett test. *P < 0.05.

**Figure 3:**
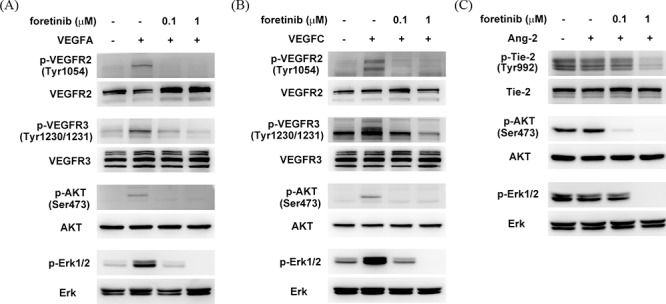
Effect of Foretinib on VEGF-A-, VEGF-C-, and ANG-2-induced activation of VEGFR-2, VEGFR-3 and TIE-2 in LECs After serum starvation for 16 h, LECs were pre-treated with Foretinib for 1 h and then stimulated with VEGF-A **A.** VEGF-C **B.** or ANG-2 **C.** for 20 min before protein extraction. The level of total or phosphorylated form of VEGFR-2, VEGFR-3, TIE-2, AKT and ERK were investigated by Western blotting.

We next investigated the effect of Foretinib on lymphangiogenesis induced by two pro-lymphangiogenic factors VEGF-C and ANG-2 because Foretinib could inhibit their cognate receptors VEGFR-3 and TIE-2. We measured the number of junctions in the tube network (number of nodes), the linear length of the tubes that connect with at least one node (tube length) and the area of tube network enclosed by tube (tube area). As shown in Fig. 2A, VEGF-C significantly increased the node number, tube length and tube area of LECs and Foretinib at the concentrations of 0.1 and 1 μM inhibited the effect of VEGF-C. In the spheroid sprout assay, spheres of LECs were embedded in a three-dimensional collagen gel and the sprouts induced by VEGF-C were counted. VEGF-C increased the sprouting of LECs spheroid and the increase was inhibited by Foretinib (Fig. 3B). ANG-2 also increased the node number, tube length and tube area of LECs which was inhibited by 1 μM of Foretinib. However, Foretinib at 0.1 μM only significantly decreased the tube area but not the node number and tube length (Fig. 2C). In the spheroid sprout assay, ANG-2 increased the number of sprouts, accumulated length per spheroid and average sprouting length and the increase was also inhibited by Foretinib (Fig. 2D).

We next tested whether Foretinib inhibited growth factor-induced activation of VEGFR-2, VEGFR-3 and TIE-2. VEGF-A and VEGF-C induced the phosphorylation of VEGFR-2, -3, AKT and ERK which was completely inhibited by 0.1 μM of Foretinib (Fig. 3A and 3B). Interestingly, phosphotylation of TIE-2, AKT and ERK was not strongly stimulated by ANG-2. This could be due to autocrine stimulation because lymphatic endothelial cells have been shown to express and release large amount of ANG-2 [19]. However, activity of TIE-2, AKT and ERK was indeed suppressed by Foretinib in a dose-dependent manner (Fig. 3C). These data suggested that Foretinib inhibited VEGF-A/C- and ANG-2-induced tube formation and spheroid and sprouting by suppressing receptor activation.

### Long-term treatment of Foretinib caused apoptosis in LECs

**Figure 4:**
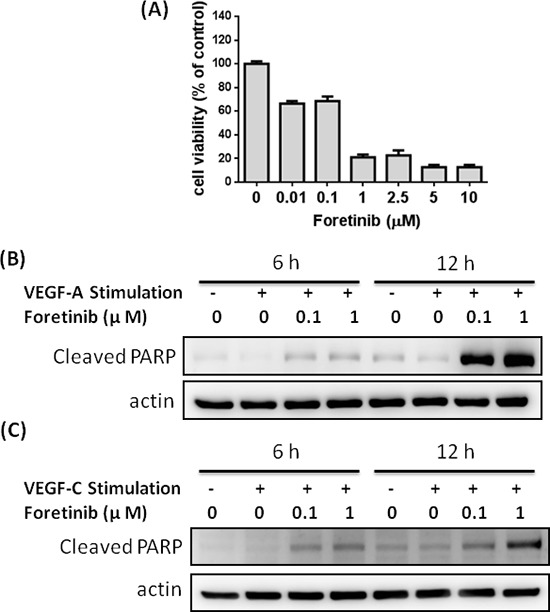
Foretinib induces apoptosis of LECs **A.** LECs were treated with indicated concentrations of Foretinib in endothelial growth medium for 48 h and the cell viability was measured. Values were Mean ± SEM of three separate experiments. **B.** LECs were treated with different combinations of VEGF-A and Foretinib for 6 or 12 h. Cellular proteins were harvested for the detection of the cleaved PARP, an apoptotic marker. **C.** LECs were treated with different combinations of VEGF-C and Foretinib for 6 or 12 h and the cleaved PARP protein was detected by Western blotting.

We also investigated LEC survival after long-term treatment of Foretinib. Our results showed that viability of LECs was dramatically reduced by 30% and 80% by 0.1 and 1 μM of Foretinib respectively (Fig. 4A). The response of LECs is much stronger than that of pancreatic cancer cell lines. As shown in Fig. 4B and 4C, no significant apoptosis was found in control and VEGF-A- or VEGF-C-treated cells as determined by the appearance of cleaved poly-ADP ribose polymerase (PARP). Foretinib significantly increased PARP degradation in a dose-dependent manner which could not be rescued by VEGF-A or VEGF-C (Fig. 4B and 4C). These data indicated that Foretinib inhibited VEGFR-2 and VEGFR-3 activity and increased apoptosis of LECs after long-term incubation.

### Foretinib inhibited tumor growth, angiogenesis and lymphangiogenesis in xenograft animals

**Figure 5:**
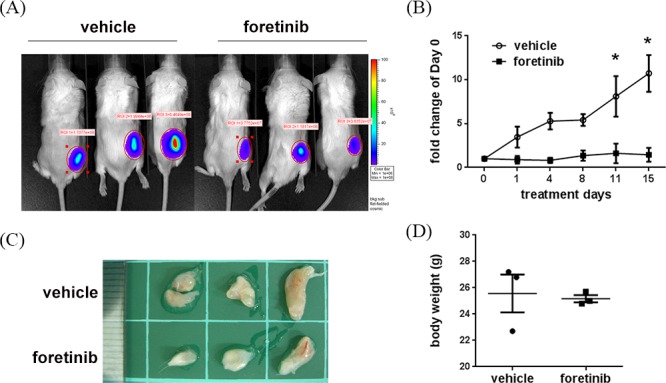
Effect of Foretinib on tumor growth in Panc-1 xenograft animals Panc-1 cells were implanted into NOD/SCID mice as described in “Materials and methods”. After two weeks, the mice were treated with vehicle or 30 mg/kg of Foretinib every day for 15 days. **A.** *In vivo* fluorescence imaging of NOD/SCID mice implanted with Panc-1 cells at day 15. **B.** Fluorescence signals of the tumors were quantified by using IVIS200 software. Values were Mean ± SEM (*n* = 3). Statistical analysis was determined by Two-Way ANOVA, followed by Bonferroni test. *P < 0.05. **C.** The tumors of the vehicle and Foretinib-treated groups. **D.** Mean of body weight of the mice at sacrifice were shown (*n* = 3).

We evaluated the anti-tumor activity of Foratinib in a xenograft animal model. Panc-1 cells with stable expression of luciferase were implanted on the right flank back and tumor growth was continuously monitored. After two weeks, the mice were randomly distributed into control group and Foretinib group and were treated by oral gavage with vehicle or Foretinib (30 mg/kg) once a day for 15 days. IVIS imaging and the photograph of tumors demonstrated that the tumor size of Foretinib group was smaller than that of control group (Fig. 5A and 5C). The quantification result of live imaging also showed that Foretinib significantly decreased the growth of Panc-1 xenograft (Fig. 5B). No significant difference of body weight was found between vehicle- and Foretinib-treated mice (Fig. 5D).

**Figure 6:**
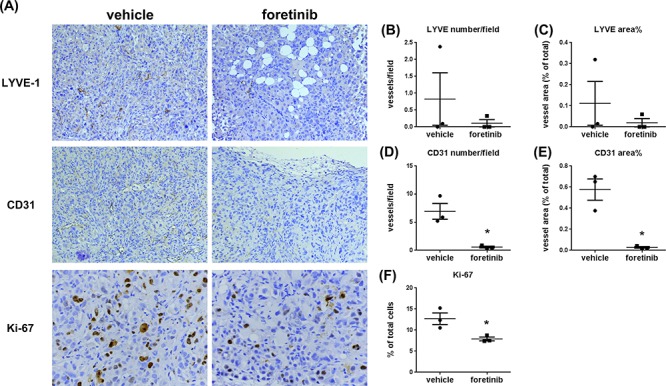
Foretinib inhibits lymphangiogenesis, angiogenesis and cell proliferation in Panc-1 xenograft animals **A.** Representative pictures of lymph vessels stained with LYVE-1 antibody, blood vessels stained with CD31 antibody and proliferative cells stained with Ki-67 antibody in vehicle- or Foretinib-treated tumor were shown. Averaged number per field and the percentage of tumor area of lymph vessels positive for LYVE-1 **B, C.** for blood vessels positive for CD31 **D, E.** and the percentage of tumor area of Ki-67-positive proliferative cells **F.** were expressed as Mean ± SEM (*n* = 3). Statistical analysis was determined by Student's *t*-test. *P < 0.05 when compared to the vehicle group.

To examine lymphangiogenesis, angiogenesis and cell proliferation in tumors, the expression of LYVE-1, CD31 and Ki-67 were analyzed by immunohistochemical assay. The representative figures of LYVE-1, CD31 and Ki-67 staining were shown in Fig. 6A. The mean of lymph vessel densities in tumors of control and Foretinib groups were 0.83 ± 0.78 and 0.11 ± 0.11, respectively (Fig. 6B). The percentage of lymph vessel area in tumors was decreased in the Foretinib group (0.11 ± 0.11% vs 0.02 ± 0.02%, *p* < 0.05) (Fig. 6C). In addition, the mean blood vessel density (6.94 ± 1.39 vs 0.59 ± 0.15, *p* < 0.05) and the percentage of blood vessel area (0.58 ± 0.10% vs 0.03 ± 0.01, *p* < 0.05) in tumors were also reduced by Foretinib (Fig. 6D–6E). Finally, we checked the proliferation marker Ki-67 in tumors. The percentage of positive Ki-67 staining of the control group was 12.72 ± 1.37% while it reduced to 7.91 ± 0.45 in Foretinib group (Fig. 6F). These data suggested that Foretinib inhibited lymphangiogenesis, angiogenesis and cell proliferation in xenograft animals.

### Foretinib suppresses pancreatic tumor growth in orthotopic animals

**Figure 7:**
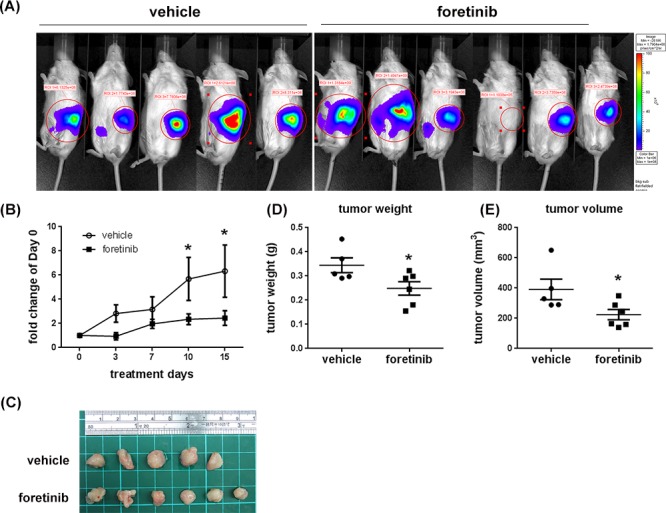
Effect of Foretinib on the growth of orthotopic tumors Panc-1 cells were implanted into the pancreas of NOD/SCID mice as described in “Materials and methods”. After 4 weeks, the mice were treated with vehicle or 30 mg/kg of Foretinib every day for 15 days by oral gavage. **A.** *In vivo* fluorescence imaging of NOD/SCID mice implanted with Panc-1 cells at day 15. **B.** Fluorescence signals of the tumors were quantified by using IVIS200 software. Values were Mean ± SEM. Statistical analysis was determined by Two-Way ANOVA, followed by Bonferroni test. *P < 0.05 when compared to the control group. **C.** The tumors harvested from the vehicle and Foretinib-treated groups. Mean of tumor weight **D.** and tumor volume **E.** of the vehicle group (*n* = 5) and the Foretinib group (*n* = 6) at sacrifice were shown. Statistical analysis was determined by Student's *t*-test. *P < 0.05 when compared to the vehicle group.

To examine the anti-tumor effect of Foretinib in the specific organ environment, we used the orthotopic animal model. After implanting Panc-1 cells into the pancreas of NOD/SCID mice for 4 weeks, the mice were treated with vehicle or Foretinib (30 mg/kg) by oral gavage once a day for 15 days. IVIS imaging and the photograph of tumors showed the tumor size was reduced in Foretinib group (Fig. 7A and 7C). The quantification of live imaging also demonstrated that Foretinib significantly reduced tumor growth (Fig. 7B). The tumor weight and tumor volume of the experimental animals at the end of treatment confirmed the IVIS imaging data (Fig. 7D and 7E).

**Figure 8:**
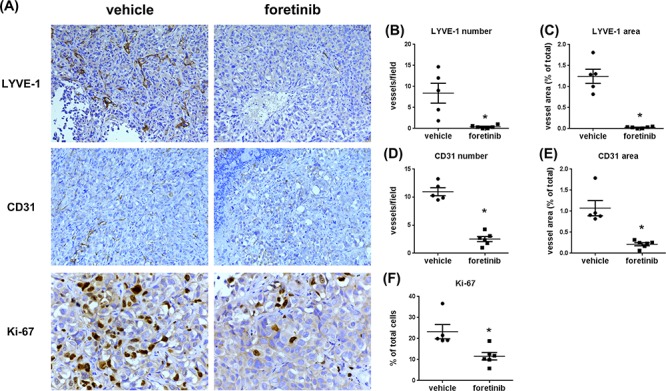
Foretinib inhibits lymphangiogenesis, angiogenesis and cell proliferation in orthotopic animals **A.** Representative pictures of lymph vessels of the tumors harvested from the pancreas of the experimental animals stained with LYVE-1 antibody, blood vessels stained with CD31 antibody and proliferative cells stained with Ki-67 antibody in vehicle- or Foretinib-treated tumor were shown. Averaged number per field and the percentage of tumor area of lymph vessels positive for LYVE-1 **B, C.** for blood vessels positive for CD31 **D, E.** and the percentage of tumor area of Ki-67-positive proliferative cells **F.** were expressed as Mean ± SEM (*n* = 3). Statistical analysis was determined by Student's *t*-test. *P < 0.05 when compared to the vehicle group.

**Figure 9:**
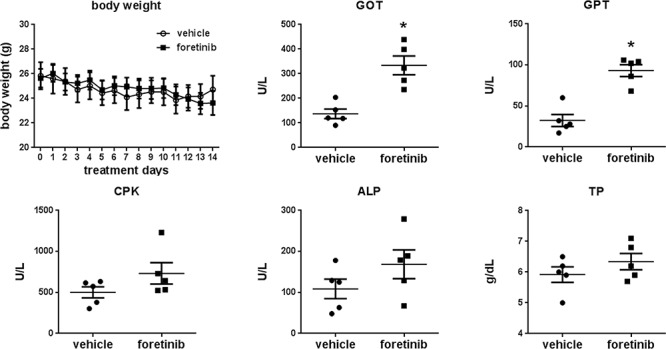
Effect of Foretinib on liver and kidney function of the orthotopic animals **A.** Body weight change of the mice of the vehicle and Foretinib groups during treatment. **B.** Blood was obtained from animals at sacrifice and serum was collected by routine procedure. The parameters of liver and kidney function of vehicle- or Foretinib-treated mice were analyzed and expressed as Mean ± SD. Statistical analysis was determined by Student's *t*-test. *P < 0.05 when compared to the vehicle group. CPK: creatinine phosphokinase; ALP: alkaline phosphatase; TP: total protein; GOT: glutamate oxaloacetate transaminase and GPT: glutamate pyruvate transaminase.

Expressions of LYVE-1, CD31 and Ki-67 were all reduced by Foretinib treatment (Fig. 8A). The mean of lymph vessel density in tumors was reduced from 8.41 ± 2.36 to 0.41 ± 0.16 and the percentage of lymph vessel area was reduced from 1.24 ± 0.17% to 0.03 ± 0.01% by Foretinib (Fig. 8B and 8C). In addition, the mean of blood vessel density and the percentage of blood vessel area in tumors were suppressed by 70–80% in Foretinib group (Fig. 8D and 8E). An averaged 50% of reduction of Ki-67-positive tumor cells was detected in Foretinib-treated tumors (Fig. 8F). The body weight of control and Foretinib groups was similar (Fig. 9). The serum levels of creatinine phosphokinase (CPK), alkaline phosphatase (ALP) and total protein (TP) were not significantly changed. However, Foretinib treatment increased serum level of glutamate oxaloacetate transaminase (GOT) and glutamate pyruvate transaminase (GPT) (Fig. 9).

## DISCUSSION

Pancreatic cancer is highly malignant and is resistant to chemotherapy and radiotherapy [8, 21, 22]. Currently, gemcitabine was used as the standard therapy of advanced pancreatic cancer. However, the response rate is low and the overall survival is only modest improved [9]. Therefore, development of new treatment strategies is urgently needed.

Because the HGF/c-MET signaling is one of the most frequently dysregulated pathways in pancreatic cancer [11, 12], targeting of this pathway received much attention recently. Crizotinib (PF-2341006) originally developed as a selective c-MET inhibitor has been tested in the therapy of various solid tumors [23]. Recent studies showed that Crizotinib is also active against anaplastic lymphoma kinase (ALK) [24, 25] and has been approved for the treatment of locally advanced or metastatic non small cell lung cancer that is ALK-positive [26]. The application of Crizotinib in pancreatic cancer is at early stage. A recent study demonstrated Crizotinib exhibits anti-tumor activity by targeting ALK but not c-MET in pancreatic cancer [27]. Another fast progressing c-MET targeting agent is Cabozantinib (XL184 or BMS907351) which inhibits c-MET, VEGFR-2, RET, KIT, FLT-3 and TIE-2 [28]. Recently, Cabozantinib has been approved for the treatment of patients with progressive medullary thyroid carcinoma [29]. Currently, a phase 1 trial in studying the safety and tolerability of the combination of Cabozantinib and gemcitabine and a phase 2 trial in treating patients with pancreatic neuroendocrine tumor are undergoing (www.clinicaltrials.gov).

Foretinib is another c-MET inhibitor under intensive investigation. In patients with recurrent or metastatic squamous cell carcinoma of the head and neck, stable disease with minor tumor shrinkage was found in half of patients received Foretinib treatment and a combinational therapy with targeted agents or cytotixic chemotherapy was suggested [30]. In patients with metastatic gastric cancer, single-agent Foretinib did not exhibit good efficacy in unselected patients although 20–23% of cases showed stable disease [31]. For papillary renal cell carcinoma, overall response rate is 13.5% [32]. An interesting observation is that a high response rate was found in patient with germline c-MET mutations [32]. Results of these phase 2 clinical trials revealed some benefits in cancer patients with a manageable toxicity. However, the effect of Foretinib on pancreatic cancer has not been evaluated. In this study, we provided the first evidence that Foretinib inhibits pancreatic cancer *in vitro* and *in vivo* by simultaneously suppressing tumor growth, angiogenesis and lymphangiogenesis. Because Foretinib only marginally reduced proliferation of pancreatic cancer *in vitro*, we suggested Foretinib suppressed tumor growth via an indirect effect by inhibiting the tumor microenvironment. A similar mechanism has also been proposed in the study of an mTOR inhibitor on breast cancer [33].

A novel finding of our study is the inhibition of lymphangiogenesis by Foretinib. Because Foretinib is a VEGFR2 inhibitor, its anti-angiogenic effect is expected and two previous studies clearly demonstrated the suppression of tumor angiogenesis in melanoma and pancreatic islet cancer animal models by this inhibitor [4, 10]. However, the anti-lymphangiogenic activity of Foretinib is unknown. The IC_50_ of Foretinib against VEGFR-2 and VEGFR-3 is similar [1]. In addition, Foretinib potently inhibited TIE-2 activity. We hypothesized that Foretinib may target these RTKs to suppress lymphangiogenesis. Our results demonstrated that Foretinib inhibited growth factor-induced tube formation and sphere sprouting of LECs and also caused apoptosis of LECs after long-term treatment. Both xenograft and orthotopic animal studies confirmed the anti-lympahangiogenic activity of Foretinib *in vivo*. Lymphangiogenesis and lymphatic vessel density strongly correlate with lymph node metastasis in pancreatic cancer [34, 35]. As shown in Fig. 8A–8C, pancreatic tumor expressed high lymphatic vessel density which could be effectively inhibited by Foretinib. Therefore, the effect of Foretinib on regional and distant metastasis of pancreatic cancer warrants further investigations.

We monitored the blood biochemical markers of liver and renal function and found the increase of GOT and GPT after Foretinib treatment. Up-regulation of these two serum markers was also reported in gastric patients of the intermittent dosing group that received 240 mg/day for 5 consecutive days every 2 weeks [31]. However, aleteration of liver function was not found in another daily dosing group. We suggested the increase of GOT and GPT in this study could be caused by dosing regimens.

Collectively, we conclude that Foretinib simultaneously targets cancer cells and LECs to inhibit pancreatic tumor growth and this multiple kinase inhibitor suppresses angiogenesis and lymphangiogenesis by blocking VEGFR-2, VEGFR-3 and TIE-2 signaling.

## MATERIALS AND METHODS

### Cell lines and reagents

Pancreatic cancer cell line Panc-1 stably expressing luciferase (Panc-1-Luci) was kindly provided by Dr. Kelvin Kun-Chih Tsai (National Institute of Cancer Research, NHRI). Cells were maintained in high glucose DMEM media containing 10% fetal bovine serum (FBS), and Penicillin/Streptomycin (Invitrogen, Carlsbad, CA) at 37°C in a humidified atmosphere with 5% CO_2_. Cells were tested for mycoplasma contamination by polymerase chain reaction. Human dermal LECs (HDLECs) were obtained from PromoCell (Heidelberg, Germany) and cultured in endothelial cell growth medium MV2 (EGM-MV2) according to manufacturer's instruction. Primary antibodies mouse anti-VEGFR-2 (A-3), rabbit anti-VEGFR-3 (C-20), rabbit anti-TIE-2 (C-20) were purchased from Santa Cruz Biotechnology (Santa Cruz, CA, USA). Rabbit anti-VEGFR-2 [T1054] phosphospecific antibody and mouse anti-β-actin were purchased from Millipore (Billerica, MA, USA). Rabbit anti-VEGFR-3 [T1230/T1231] phosphospecific antibody was purchased from Cell Application (San Diego, CA, USA). Rabbit anti-TIE-2 phosphospecific antibody was purchased from R&D (Abingdon, UK). Rabbit anti-AKT total or phosphospecific [S473] antibody, rabbit anti-ERK1/2 total and phosphospecific [T202/Y204] antibody were obtained from Cell Signaling Technology (Danvers, MA, U.S.A.). Rabbit anti-LYVE-1 and rabbit anti-CD31 antibody were obtained from Abcam (Cambridge, MA, USA). Mouse anti-Ki-67 (SP6) antibody was obtained from Spring Bioscience Corp (Pleasanton, CA, USA). Foretinib was purchased from APExBIO (Houston, TX, USA). Stock solutions were prepared in dimethyl sulfoxide (DMSO as vehicle) and stored in aliquots at −80°C. VEGF-A, VEGF-C and ANG-2 recombinant proteins were purchased from R&D (Abingdon, UK).

### Growth assay

Panc-1-Luci cells were seeded onto 24-well plates at a density of 10000 cells/well and incubated in growth medium. For LECs, cells were seeded onto 24-well plates at a density of 15000 cells/well. After 24 h, the medium was changed into growth medium containing different concentrations of Foretinib and incubated for 24 or 48 h. At indicated time points, cells were pre-incubated with 50 μl/well of 5 mg/ml 3-(4, 5-dimethylthiazol-2-yl)-2, 5-diphenyltetrazolium bromide (MTT) for 4 h and optical density was read at 570 nm by using a ELISA reader. All experiments were done in triplicates at least three times.

### Tube formation assay

For tube formation assay, 10 μl of ice cold, growth factor-reduced matrigel was added to a pre-chilled channel slide and allowed gelation at 37°C for 30 min. LECs were pre-incubated with 0, 0.1 or 1 μM Foretinib for 20 min. The cells were treated with 10 ng/ml VEGF-A or 100 ng/ml VEGF-C and seeded into plates at the density of 6 × 10^3^ cells/well. After 4 h, the images of tube formation were taken by using a Leica DMI 4000 phase-contrast microscope (Leica Microsystems, Germany) with contrast objective. Tube length, area and nodule number were quantified by using the NIH *ImageJ software*.

### Sprouting assay

To generate multicellular spheroids, LEC cells were suspended in EGM medium containing 0.24% high viscosity methyl cellulose (Sigma Aldrich, Saint Louis, MO) Cells (1 × 10^3^ cells per drop) were seeded onto the lids of tissue culture plates. After 24 h, the spheroids were collected and embedded in collagen gels (3 mg/ml acidic collagen, 10× HBSS, PBS and 0.1 N NaOH with a volume ratio of 5:3:3:1, pH7.4). The gel containing spheroid was transferred into channel slides and allowed gelation at 37°C for 30 min. Medium containing growth factors and Foretinib was added on top of the gels and the gels were incubated at 37°C. After 24 h, the images were taken by using a Leica DMI 4000 phase-contrast microscope (Leica Microsystems, Germany). The length and number of the sprouts were quantified by using the NIH *ImageJ* software and at least 20 spheroids were analyzed per experimental group.

### Western blotting

The cells were lysed in RIPA buffer containing Halt protease and phosphatase inhibitor cocktail (Thermo Scientific). Protein concentration was measured by BCA protein assay kit (Thermo Scientific, Schwerte, Germany). Thirty μg of the lysates were resolved by SDS-PAGE and transferred to PVDF membranes. Membranes were blocked with 5% nonfat milk for 1 h, and then incubated with primary antibodies in blocking buffer at 4°C overnight. Goat anti-rabbit or rabbit anti-mouse IgG conjugated with HRP were used as secondary antibodies. Detection was enhanced by chemiluminescence, and signal was detected by UVP Imaging System.

### Immunohistochemistry

Paraffin embedded tissues were sectioned, deparaffinized and rehydrated. Tissue sections were heated by micro-wave (640 Watt) in 10 mM citrate buffer, pH 6.0, for 15 min, and then rinsed three times in PBS. The sections were quenched of endogenous peroxidase activity with 3% H_2_O_2_ in PBS at room temperature for 10 min and followed by blocking in 3% BSA in PBST. The sections were incubated with primary antibodies overnight at 4°C. The signal was developed by using the Mouse/rabbit polydetector HRP/DAB detection system (BioSB, Santa Barbara, CA, USA) and the slides were counterstained with hematoxylin. Non-specific staining was assessed by omission of the primary antibody as well as replacing primary antibody with non-immunized rabbit or mouse serum.

### Animal experiments

NOD/SCID mice were purchased from NCKU laboratory animal center (Tainan, Taiwan). For xenograft animal model, ten-week old mice were injected subcutaneously with 2 × 10^6^ Panc-1-Luci cells suspended in 100 μl of HBSS containing 50% Matrigel by using a 25-gauge needle after deep anesthesia was induced. After 2 weeks, mice received vehicle or Foretinib (30 mg/kg, suspended in 1% hydroxypropylmethylcellulose and 0.2% sodium *dodecyl* sulfate in H_2_O) by oral gavage for 15 days. For orthotopic animal model, Panc-1-Luci cells (1 × 10^6^ cells suspended in 50 μl HBSS) were injected into pancreas of mice. After 4 weeks, mice received the dosing of Foretinib as described in xenograft animl model. The mice were imaged by the IVIS imaging system twice per week until the tumors were harvested. For *in vivo* imaging, mice were injected with d-luciferin (150 mg/kg in saline) by intraperitoneal injection immediately after administration of anesthesia with isoflurane. After administration of d-luciferin for 2 min, the anesthetized mice were placed onto the light-tight camera box with continuous exposure to isoflurane. The IVIS camera system was used to visualize the tumors, and photon measurement was defined around the tumor area and quantified as total photon by using Living Image software (Xenogen, Corp, Alameda, CA).

### Analysis of blood chemistry

Blood was obtained from orthotopic animals at sacrifice and serum was collected by routine procedure. The parameters of liver and kidney function were analyzed by Fuji Dri-Chem 4000i autoanalyzer of the Taiwan Mouse Clinic (Taipei, Taiwan).

### Statistical analysis

Appropriate statistical analyses were performed by using Prism 5 (Version 5.04, Graphpad Software, Inc., La Jolla, CA, USA). For statistical analyses, Student's *t*-test, one-way ANOVA and two-way ANOVA were used. A *p* value less than 0.05 was considered statistically significant.
